# Oxidative stress response in regulatory and conventional T cells: a comparison between patients with chronic coronary syndrome and healthy subjects

**DOI:** 10.1186/s12967-021-02906-2

**Published:** 2021-06-03

**Authors:** Anna K. Lundberg, Rosanna W. S. Chung, Louise Zeijlon, Gustav Fernström, Lena Jonasson

**Affiliations:** 1 Department of Health, Medicine and Caring Sciences, Unit of Cardiovascular Medicine, Linköping University, Linköping, Sweden; 2 Department of Cardiology in Linköping, and Department of Health, Medicine and Caring Sciences, Unit of Cardiovascular Medicine, Linköping University, Linköping, Sweden; 3 Department of Cardiology, Linköping University Hospital, 581 85 Linköping, Sweden

**Keywords:** Coronary artery disease, Chronic coronary syndrome, T cell, Regulatory T cell, Oxidative stress

## Abstract

**Background:**

Inflammation and oxidative stress form a vicious circle in atherosclerosis. Oxidative stress can have detrimental effects on T cells. A unique subset of CD4^+^ T cells, known as regulatory T (T_reg_) cells, has been associated with atheroprotective effects. Reduced numbers of T_reg_ cells is a consistent finding in patients with chronic coronary syndrome (CCS). However, it is unclear to what extent these cells are sensitive to oxidative stress. In this pilot study, we tested the hypothesis that oxidative stress might be a potential contributor to the T_reg_ cell deficit in CCS patients.

**Methods:**

Thirty patients with CCS and 24 healthy controls were included. T_reg_ (CD4^+^CD25^+^CD127^−^) and conventional T (CD4^+^CD25^−^, T_conv_) cells were isolated and treated with increasing doses of H_2_O_2_. Intracellular ROS levels and cell death were measured after 2 and 18 h, respectively. The expression of antioxidant genes was measured in freshly isolated T_reg_ and T_conv_ cells. Also, total antioxidant capacity (TAC) was measured in fresh peripheral blood mononuclear cells, and oxidized (ox) LDL/LDL ratios were determined in plasma.

**Results:**

At all doses of H_2_O_2,_ T_reg_ cells accumulated more ROS and exhibited higher rates of death than their T_conv_ counterparts, p < 0.0001. T_reg_ cells also expressed higher levels of antioxidant genes, including thioredoxin and thioredoxin reductase-1 (p < 0.0001), though without any differences between CCS patients and controls. T_conv_ cells from CCS patients were, on the other hand, more sensitive to oxidative stress ex vivo and expressed more thioredoxin reductase-1 than T_conv_ cells from controls, p < 0.05. Also, TAC levels were lower in patients, 0.97 vs 1.53 UAE/100 µg, p = 0.001, while oxLDL/LDL ratios were higher, 29 vs 22, p = 0.006.

**Conclusion:**

T_reg_ cells isolated from either CCS patients or healthy controls were all highly sensitive to oxidative stress ex vivo. There were signs of oxidant-antioxidant imbalance in CCS patients and we thus assume that oxidative stress may play a role in the reduction of T_reg_ cells in vivo.

**Supplementary Information:**

The online version contains supplementary material available at 10.1186/s12967-021-02906-2.

## Background

Oxidative stress and chronic inflammation are closely linked phenomena that can perpetuate each other and easily form a vicious circle in chronic disease. The simultaneous existence of oxidative stress and low-grade inflammation in atherosclerosis is well established. Oxidative stress, defined as chronic overproduction of reactive oxygen species (ROS) or rather an imbalance between oxidants and antioxidants in favor of the oxidants [1], plays a major role in the development of atherosclerosis [2, 3]. In humans, a variety of circulating markers of oxidative stress, including oxidative modification of low density lipoprotein (LDL), have shown associations with cardiovascular disease [4, 5]. It has also been shown that oxidized (ox) LDL can elicit a robust immune response in atherosclerosis, involving activation of CD4^+^ T cells [6].

Although moderate levels of ROS are necessary for the proper regulation of T cell activation, large quantities of ROS may have detrimental effects on T cells, such as decreased viability [7]. This may be an area of special interest in understanding the T cell perturbations associated with coronary artery disease. The systemic T cell activation that occurs in many patients with acute coronary syndrome (ACS) does not normalize over time but becomes persistent, despite clinical stability and medical treatment [8–10].

The perturbed T cell repertoire in patients with coronary artery disease is also associated with a decrease of regulatory T (T_reg_) cells, a specialized subpopulation of CD4^+^CD25^+^ T cells. These cells were first identified in 1995 by Sakaguchi et al. [11] as a subset of thymus-derived CD4^+^ T cells that expressed high levels of the interleukin (IL-2) receptor α-chain CD25 and protected thymectomized mice from autoimmunity. In humans, several autoimmune and inflammatory pathologies have been associated with numerical deficits in T_reg_ cells as well as increased apoptosis of T_reg_ cells [12–14]. Interestingly, T_reg_ cell deficits have also been reported in patients with ACS as well as in those with CCS, the latter a term defining patients with stable coronary artery disease [9, 15–17]. Whether T_reg_ cells can function as new targets in atherosclerotic disease has become a subject of increasing interest [18]. However, it is still unclear why T_reg_ cells are reduced in patients with coronary artery disease but one intriguing possibility is that oxidative stress plays a major role. In a previous study, Mor et al. [16] demonstrated that the numbers of CD4^+^CD25^+^ T_reg_ cells were markedly reduced after incubation with oxLDL while the effect on conventional CD4^+^CD25^−^ T (T_conv_) cells was negligible. The finding indicating that T_reg_ cells were more sensitive to oxidative stress compared to T_conv_ cells was however contradicted by Mougiakakos et al. [19] who showed that T_reg_ cells from healthy volunteers were more resistant to hydrogen peroxide (H_2_O_2_)-induced cell death compared to T_conv_ cells.

The extent to which oxidative stress affects T_reg_ cell survival in coronary artery disease is thus far from clarified. In this pilot study, we tested the hypothesis that oxidative stress is a contributor to the T_reg_ cell deficit in CCS patients. Our first aim was to perform a comparison between T_reg_ and T_conv_ cells with respect to sensitivity towards oxidative stress. A second aim was to investigate whether T_reg_ and T_conv_ cells from CCS patients and healthy controls differed in their sensitivity towards oxidative stress. Different aspects of oxidative stress response were examined in freshly isolated T_reg_ and T_conv_ cells. We studied the sensitivity to oxidative stress-induced cell death ex vivo by treating cells with H_2_O_2_, a major member of the ROS family. We also determined the impact of H_2_O_2_ on cellular oxidative stress by measuring total (cytoplasmic and nuclear) cellular ROS at the single cell level. To further elucidate the oxidative stress response in vivo, we measured the expression and secretion of endogenous antioxidants in T_reg_ and T_conv_ cells from patients and controls.

## Methods

### Study population

The study population consisted of patients (n = 30) recruited from the Department of Cardiology, University Hospital, Linköping, Sweden, as well as control subjects with approximately equal sex and age distribution (n = 24). All patients had significant coronary artery disease, defined as ≥ 50% luminal narrowing in any of the major epicardial coronary arteries (i.e., the right coronary artery, left anterior descending artery, and left circumflex artery), and a history of coronary event, i.e. ACS or stable angina followed by coronary revascularization. For the control group, individual residents of Linköping were randomly selected from the Swedish Population Register and invited to participate in the study. Individuals who accepted the invitation were included as controls if they were anamnestically healthy and had normal routine laboratory tests. Use of lipid-lowering or antihypertensive drugs for primary prevention of cardiovascular disease was allowed in the control group.

Study subjects were excluded if they suffered from severe heart failure, immunological disorders, neoplastic disease, had evidence of acute or recent (< 2 months) infection or major trauma, had undergone surgery/revascularization procedure (< 2 months) or received regular treatment with immunosuppressive or anti-inflammatory agents (except low-dose aspirin).

### Cell isolation

Peripheral blood mononuclear cells (PBMCs) were isolated from diluted sodium heparinized whole blood using Ficoll-Paque Density Gradient Medium (ThermoFisher Scientific) as previously described [20]. An EasySep Human CD4^+^CD127^low^CD25^+^ Regulatory T Cell Isolation Kit (STEMCELL Technologies) was used according to manufacturer´s instructions on PBMCs resulting in two cell fractions; T_reg_ cells defined as CD4^+^CD127^low^CD25^+^ and T_conv_ cells defined as CD4^+^CD127^+^CD25^−^ T cells. After isolation cells were resuspended in complete RPMI (Fisher Scientific) with 10% FBS (Fisher Scientific) and 2% Penicillin Streptomycin solution (Fisher Scientific).

### Purity checks

For purity checks, cells were stained with a Human Regulatory T Cell Sorting Kit cocktail including CD45RA-FITC, CD127-Alexa Fluor647, CD25-PE, and CD4-PerCP-Cy5.5 (BD Biosciences) and analysed on a FACS aria (BD Biosciences). Cells were separately stained for FoxP3 expression using a Human FoxP3 Buffer Set (BD Biosciences) together with antibodies against CD4-FITC and FoxP3-V450 and analysed with Gallios flow cytometer (Beckman Coulter). Illustrative results are shown in Additional file 1: Figure S1.

### H_2_O_2_-induced cell death of T_reg_ and T_conv_ cells

A total of 40,000 T_reg_ or T_conv_ cells were treated with 5, 10, 20, or 30 μM H_2_O_2_ or left untreated in a total volume of 200 μL in a round-bottom 96-well plate. Cells were incubated for 18 h in a humified incubator at 37 °C and 5% CO_2_. The incubation time was chosen based on previous similar studies [19, 21]. Following incubation, cells were washed and 100 µL Annexin-V Binding Buffer (BD Biosciences), Annexin-V PE and CD4-BV510 antibodies (BD Biosciences) was added. After 15 min 400 µL Annexin-V Binding Buffer and SYTOX Red Dead Cell Stain (ThermoFisher) was added. After another 10 min, cells were analyzed within 1 h with a Gallios flow cytometer to monitor cell death.

### Staurosporin-induced cell apoptosis of Tregand T_conv_ cells

In the same manner as described above, T_reg_ and T_conv_ cell fractions were also treated with 2.5 µM staurosporin (STS) (Streptomyces sp. Origin, Sigma-Aldrich) as an alternative way to induce apoptosis.

### H_2_O_2_-induced intracellular ROS levels in T_reg_ and T_conv_ cells

A total of 40 000 T_reg_ or T_conv_ cells were treated with 30 µM, 60 µM and 120 µM H_2_O_2_ at 37 °C for a total of 2 h. After 1 h, CellROX Green (Thermofisher), a measure of total (cytoplasmic and nuclear) cellular ROS, was added. Thereafter cells were washed with PBS + O.5% FBS and and resuspended in 100 µL PBS + 0.5% FBS and SYTOX Red Dead Cell Stain, CD4BV510 and 10 µL Brilliant Stain Buffer (BD Biosciences) were added and kept for 15 in in the dark in room temperature. Finally, mean fluorescence intensity (MFI) of CellROX Green was recorded with a 3-laser Gallios Flow Cytometer.

### Flow cytometry analyses

Flow cytometry data were analyzed with Kaluza Analysis Software 2.1 (Beckman Coulter). Lymphocytes were gated according to size and granularity and thereafter CD4^+^ cells only were gated. Annexin-V identified early apoptosis while SYTOX identified late apoptosis and necrosis. An illustrative example is shown in Additional file 2: Figure S2. Cells negative for Annexin-V and SYTOX were considered viable. For the oxidative stress assay, cells were gated in a lymphocyte gate and the CellROX Green signal was recorded in CD4^+^ cells that were negative for SYTOX, i.e. living cells.

### Oxidative stress gene expression in circulating T_reg_ and T_conv_ cells

mRNA expression of oxidative stress-associated genes was assessed in T_reg_ and T_conv_ cells isolated from PBMCs using the same kit from STEMCELL as described above. Cell lysates were collected directly after isolation. Total RNA was isolated using Qiagen total RNA isolation kit (Thermofisher). The RNA (total 21,6 ng) was converted to cDNA using high capacity cDNA reverse transcription kit with an RNAse inhibitor (Life Technologies). cDNA was amplified by RT-PCR reactions with TaqMan™ Fast Universal PCR Mastermix (Life Technologies) on an ABI 7500 Sequence Detector (Applied Biosystems). The following TaqMan Gene Expression Assay kits were used: Hs00167309_m1 for superoxide dismutase 2 (SOD2), Hs00156308_m1 for catalase (CAT), Hs00757844_m1 for oxidation resistance 1 (OXR1), Hs04194449_s1 for glutathione peroxidase-7 (GPX7), Hs00828652_m1 for thioredoxin (Trx), Hs00917067_m1 thioredoxin reductase (TrxR1). Eukaryotic 18S rRNA (Part number: 4352930E) served as endogenous control. The expression of all genes was calculated with the comparative CT method where the amount of target, normalized to an endogenous reference and relative to a calibrator, is given by 2^ΔΔCT^ according to the user bulletin no 2 (Applied Biosystems). Results are presented as arbitrary units. Each sample was run in duplicates and a maximum deviation of 10% was allowed.

### TrxR1 in cell supernatants

The concentration of TrxR1 in cell supernatants from T_reg_ and T_conv_ cells after treatment with or without 30 μM H_2_O_2_ for 18 h was measured with an ELISA kit (Abcam, UK) according to manufacturer´s instructions. The range of the standard curve was 0.196–25 ng/mL. Samples were assayed in duplicates and a maximal deviation of 15% was allowed. Undetected samples were given half the value of the lowest standard point.

### Total antioxidant capacity in PBMCs

Total antioxidant capacity (TAC) in PBMCs was measured using Cell Biolabs, Inc. OxiSelect Total Antioxidant Capacity Assay Kit. In brief, cell pellets of 3 millions PBMCs were snap frozen and stored in − 80 °C for no longer than 2 weeks. Frozen PBMCs were lysed by thawing on ice followed by water-bath sonication for 10 min and thereafter kept on ice. The TAC assays were then performed according to manufacturer’s instructions. Results are expressed as units of uric acid equivalents (UAE).The TAC results were also normalized by protein amount measured in PBMCs using Thermo Scientific’s Coomassie Plus (Bradford) Assay Kit according to the manufacturer’s instruction. The final TAC results were calculated according to the equation below:

$$\text{TAC} = (\text{UAE}/100\,\upmu\text{g}) =\frac{UAE}{Protein\, Amount\, Assay\, (\upmu \text{g})}\times 100$$

### Oxidized LDL cholesterol in plasma

OxLDL in EDTA plasma was measured by an ELISA (Mercodia, Uppsala, Sweden), which is a capture ELISA using the mAb-4E6 antibody against a conformational epitope in oxidized ApoB-100, developed by Holvoet et al.[22]. The inter-assay variation was below 5% for the oxLDL ELISA. All samples were within the range of detection. The oxLDL/LDL ratio was used as an estimate of in vivo LDL oxidation [23].

### Statistics

Statistical analyses were calculated in IBM SPSS Statistics 25. Groups and independent variables were compared using independent Mann–Whitney U tests, Fisher´s exact test, and a Chi Square test. Wilcoxon rank sum test or paired t-test were used to compare paired data. Correlations were calculated using Spearman rank correlation test. Numeric data are presented as median and inter-quartile range. A p value < 0.05 was considered significant. Sample size calculation was based on a previous study showing that natural killer cells in CCS patients were more sensitive to apoptosis induced by oxidized lipids compared to natural killer cells in healthy controls [24].

## Results

### Characteristics of study population

The basal characteristics of the study population are presented in Table 1. There were no significant differences between CCS patients and controls with respect to age, gender, body mass index, current smoking, use of anti-hypertensive medication or laboratory variables, including high density lipoprotein (HDL) cholesterol, triglycerides, creatinine or fasting glucose levels. The prevalence of former smoking was higher among patients. Seven (23%) patients had type 2 diabetes. Twenty-three (77%) patients had a history of ACS. As regards coronary revascularization, 19 (63%) patients had a history of percutaneous coronary intervention (PCI), 15 (50%) had a history of coronary artery bypass grafting (CABG) while 4 (13%) had a history of previous PCI followed by CABG. Statins were used by all patients (except for one) resulting in significantly lower LDL levels in this group. Also, the levels of oxLDL were lower in patients. However, the oxLDL/LDL ratios were significantly higher indicating an environment of oxidative stress in the patient group. Moreover, the TAC levels in PBMCs were significantly lower in patients compared to controls, *p* = 0.001. When the 7 patients with diabetes were excluded, the TAC levels were still significantly lower in patients, *p* = 0.002.

**Table 1:** Basal characteristics of CCS patients and controls

	CCS patients (n = 30)	Controls (n = 24)	*p*
Age, years	67 (62–73)	73 (67–75)	0.053
Males	23 (77)	17 (71)	0.429
Body mass index, kg/m^2^	27 (24–30)	25 (24–28)	0.192
Smoking			
Current	1 (3.3)	0 (0)	0.556
Former	15 (50)	3 (13)	**0.004**
History of coronary event, ACS/SA	23 (77)/7 (23)	-	
History of coronary revascularization, PCI/CABG^a^	19 (63)/15 (50)	-	
Severity of coronary artery disease, 1VD/2VD/3VD^b^	7 (23)/9 (30)/14 (47)	-	
Use of anti-hypertensive drugs	14 (47)	8 (33)	0.239
Use of statin	29 (97)	7 (29)	**< 0.0001**
LDL cholesterol, mmol/L	1.6 (1.4–1.9)	3 (2.2–3.5)	**< 0.0001**
oxLDL cholesterol, U/L	43 (38–53)	68 (55–75)	**< 0.0001**
oxLDL/LDL ratio	29 (24–35)	22 (20–27)	**0.006**
HDL cholesterol, mmol/L	1.4 (1.0–1.7)	1,4 (1.0–1.7)	0.662
Triglycerides, mmol/L	1.0 (0.7–1.5)	1.3 (0.8–1.6)	0.398
Creatinine, µmol/L	88 (78–97)	79 (73–92)	0.134
Fasting glucose, mmol/L	5.8 (5.3–6.6)	5.7 (5.2–6.3)	0.403
TAC in PBMCs, UAE/100 ug	0.97 (0.28–1.48)	1.53 (1.20–2.09)	**0.001**

Values are given as n (%) or median (interquartile range). CCS: chronic coronary syndrome; ACS: acute coronary syndrome; SA: stable angina; PCI: percutaneous coronary intervention; CABG; coronary artery bypass grafting; VD: vessel disease; LDL: low density lipoprotein; oxLDL: oxidized LDL; HDL: high density lipoprotein; TAC: total antioxidant capacity; PBMC: peripheral blood mononuclear cell; UAE: uric acid equivalents

^a^ Four patients had a previous history of PCI followed by CABG

^b^ Severity of coronary artery disease is indicated as ≥ 50% luminal narrowing in one, two or three major epicardial coronary arteries (i.e., the right coronary artery, left anterior descending artery, and left circumflex artery). Bolded *P*-values represent statistical significance

### H_2_O_2_- and STS-induced cell death of T_reg_ and T_conv_ cell

**Fig. 1:**
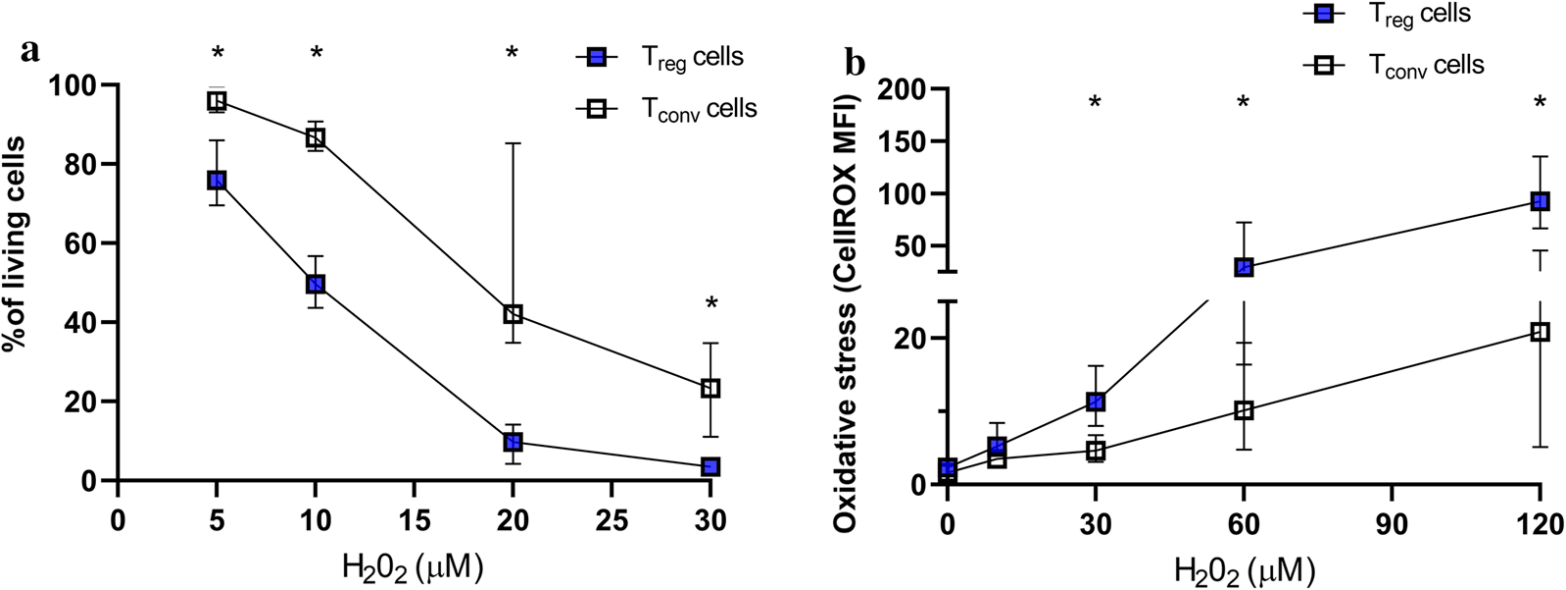
The response to oxidative stress ex vivo in T_reg_ and T_conv_ cells. **A** Percentages of living T_reg_ and T_conv_ cells, following treatment with 5, 10, 20, or 30 µM H_2_O_2_ for 18 h. % of living cells is defined as negative for Annexin-V and SYTOX and normalized based on untreated cells (medium only). Asterisks indicate significant differences between T_reg_ and T_conv_ cells, all *p* < 0.0001. Number of study subjects per dose of H_2_O_2_ were n = 19 for 5 µM, n = 33 for 10 µM, n = 22 for 20 µM, n = 34 for 30 µM. **B** The levels of oxidative stress, measured as mean fluorescence intensity (MFI) of CellROX Green, is shown in T_reg_ and T_conv_ cells following treatment with 0, 10, 30, 60 or 120 µM H_2_O_2_ for 2 h (B). Asterisks indicate significant differences between T_reg_ and T_conv_ cells, all p < 0.0001 (except for 60 µM H_2_O_2_, *p* < 0.001). Number of study subjects per dose of H_2_O_2_ were n = 22 for 0 µM, n = 3 for 10 µM, n = 20 for 30 µM, n = 20 for 60 µM, n = 12 for 120 µM. Box plots display median and i-q range values

T_reg_ and T_conv_ cells were isolated from 34 subjects (20 CCS patients and 14 controls). Among patients, 16 had a history of ACS (7 PCI, 9 CABG, 4 PCI followed by CABG) and 4 had a history of stable angina (2 PCI, 2 CABG). Separate fractions of T_reg_ and T_conv_ cells were treated with medium only or increasing concentrations of H_2_O_2_. Before treatment, the viability was > 90% in both cell types. After 18 h incubation with medium only, the proportions of living T_reg_ cells were significantly lower compared with T_conv_ cells, 71 (64–76) % vs 90 (86–93) %, p < 0.001. At all concentrations of H_2_O_2_, T_reg_ cells were more susceptible to H_2_O_2_-induced cell death than T_conv_ cells (Fig. 1A). There was a clear dose–response relationship with very low percentages of living T_reg_ cells at 20 and 30 μM H_2_O_2_.

T_reg_ cells were also more susceptible to cell death compared to T_conv_ cells when STS, a classical inducer of the intrinsic apoptotic pathway, was used. The proportions of living cells were 46 (37–54) % and 88 (79–95) %, p < 0.001, for T_reg_ and T_conv_ cells, respectively, after treatment with 2.5 µM STS.

There were no significant differences in susceptibility to H_2_O_2_-induced cell death between T_reg_ cells from CCS patients and T_reg_ cells from controls at any concentration of H_2_O_2_ (Table 2). Neither were there any differences in STS-induced cell death in T_reg_ cells between patients and controls. On the other hand, the percentages of living T_conv_ cells were significantly lower in patients than in controls when T_conv_ cells were exposed to H_2_O_2_ at lower concentrations (5 or 10 µM) or to STS (Table 2).

**Table 2:** Percentages of living regulatory T (T_reg_) cells and corresponding conventional T (T_conv_) cells from CCS patients and controls, defined as negative for Annexin-V and SYTOX and normalized based on untreated cells (medium only), after 18 h treatment with different concentrations of H_2_O_2_ or 2,5 µM STS

Treatment	CCS patients (n = 20)	Controls (n = 14)	*p*
	T_reg_ cells, % living cells		
5 µM H_2_O_2_	74 (69–88)	88 (77–94)	0.246
10 µM H_2_O_2_	50 (44–58)	58 (41–79)	0.338
20 µM H_2_O_2_	9.6 (2.8–11)	14 (8.8–29)	0.159
30 µM H_2_O_2_	2.7 (2.2–4.2)	3.9 (2.8–8.9)	0.106
2.5 µM STS	42 (30–52)	47 (40–56)	0.174

	T_conv_ cells, % living cells		
5 µM H_2_O_2_	94 (92–99)	98 (97–100)	**0.046**
10 µM H_2_O_2_	85 (92–99)	93 (87–98)	**0.030**
20 µM H_2_O_2_	39 (35–67)	69 (45–84)	0.110
30 µM H_2_O_2_	24 (11–41)	39 (21–57)	0.199
2.5 µM STS	80 (69–91)	91 (88–95)	**0.014**

Values are given as median (inter-quartile range). CCS, chronic coronary syndrome, H_2_O_2_: hydrogen peroxide; STS: staurosporin; Bolded *P*-values represent statistical significance

### H_2_O_2_-induced intracellular ROS accumulation in T_reg_ and T_conv_ cells

H_2_O_2_-induced intracellular ROS levels were measured in T_reg_ and T_conv_ cells after 2 h treatment in medium with or without increasing concentrations of H_2_O_2_. Treatment with medium only or with 10 µM H_2_O_2_ did not reveal any differences between T_reg_ and T_conv_ cells regardless of subject types while the ROS accumulation was significantly larger in T_reg_ cells treated with 30 µM H_2_O_2_ or more (Fig. 1B). When we compared CCS patients and controls, the ROS production in T_reg_ and T_conv_ cells did not show any significant differences (Additional file 3: Figures S3A and B).

### The in vivo expression of oxidative stress-associated genes in T_reg_ and T_conv_ cells

**Fig. 2:**
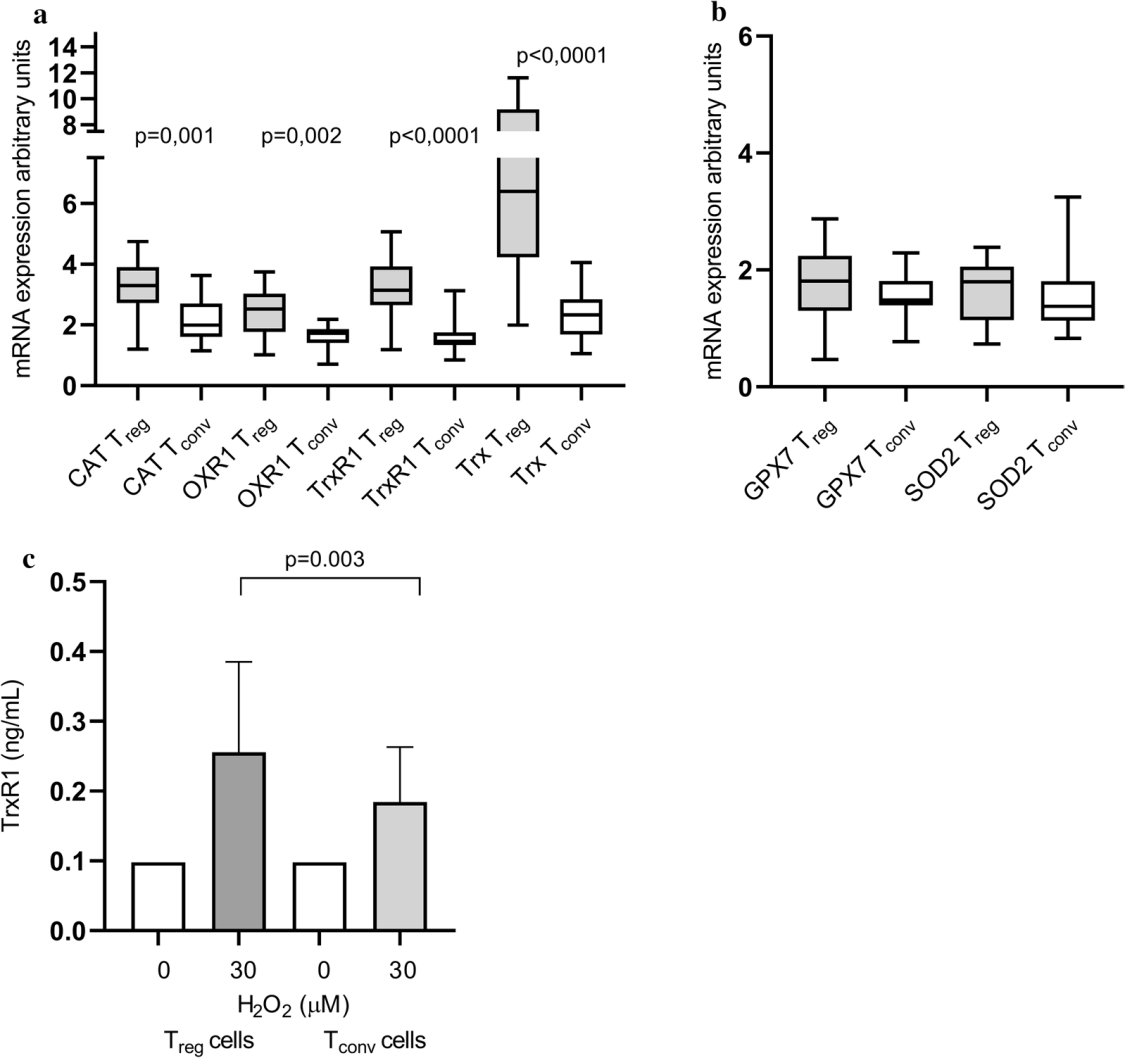
The expression of oxidative stress-associated genes in T_reg_ and T_conv_ cells from 20 study subjects (10 CCS patients and 10 controls). **A** Genes with significant differences between patients and controls included catalase (CAT), oxidation resistance 1 (OXR1), thioredoxin reductase-1 (TrxR1) and thioredoxin (Trx). **B** Genes with non-significant differences included glutathione peroxidase 7 (GPX7) and superoxide dismutase 2 (SOD2). Graphs depict arbitrary values normalized against internal control and 18S rRNA in each individual sample. **C** Thioredoxin reductase 1 (TrxR1) (ng/mL) levels in cell supernatants of T_reg_ and T_conv_ cells treated with either 0 or 30 µM H_2_O_2_. Number of study subjects per dose of H_2_O_2_ were n = 32 for 0 µM and n = 32 for 30 µM. In all figures, box plots and bars display median and i-q range values’

In order to compare the oxidative stress response in vivo, we measured the expression of a number of oxidative stress-associated genes in freshly isolated T_reg_ and T_conv_ cells from 20 subjects, 10 CCS patients (8 males, 2 females, median age 67, 7 with a previous history of ACS and 4 with a history of CABG) and 10 controls (8 males, 2 females, median age 68). As shown in Fig. 2A, the mRNA levels of CAT, OXR1, Trx and TrxR1 were significantly higher in T_reg_ cells than in T_conv_ cells regardless of subject types while mRNA levels of GPX7 and SOD2 expression did not differ between the two T cell fractions (Fig. 2B).

When comparing CCS patients and controls, the T_reg_ cells did not show any significant differences in gene expression (Table 3). On the other hand, the T_conv_ cells from CCS patients showed significantly higher expression of TrxR1 than those from controls (p = 0.033). Also, the Trx and SOD2 mRNA levels tended to be higher in T_conv_ cells from patients (p = 0.104 and p = 0.091, respectively).

**Table 3:** The mRNA expression of thioredoxin reductase-1 (TrxR1), thioredoxin (Trx), glutathione peroxidase 7 (GPX7), oxidation resistance 1 (OXR1), catalase (CAT) and superoxide dismutase 2 (SOD2) in regulatory T (T_reg_) cells and corresponding conventional T (T_conv_) cells from CCS patients and controls

	CCS patients (n = 10)	Controls (n = 10)	
Gene	T_reg_ cells		*P*
TrxR1	3.7 (2.6–4.4)	3.0 (2.6–3.9)	0.973
Trx	7.9 (4.6–10)	6.2 (4.0–6.7)	0.445
GPX7	2.0 (1.2–2.2)	1.6 (1.3–2.4)	0.621
OXR1	2.9 (1.8–3.3)	2.5 (1.7–2.7)	0.681
CAT	3.6 (2.4–4.0)	3.1 (2.8–3.9)	0.929
SOD2	1.9 (1.1–2.0)	1.7 (1.2–2.1)	0.949

	T_conv_ cells		
TrxR1	1.7 (1.4–2.0)	1.4 (1.0–1.6)	**0.033**
Trx	2.8 (2.2–3.2)	1.9 (1.5–2.4)	0.104
GPX7	1.4 (1.4–2.1)	1.5 (1.4–1.7)	0.267
OXR1	1.8 (1.4–1.9)	1.7 (1.4–1.9)	0.601
CAT	2.4 (1.9–2.7)	1.7 (1.4–2.8)	0.204
SOD2	1.6 (1.4–2.1)	1.3 (0.8–1.5)	0.091

Values are given as median (interquartile range). CCS, chronic coronary syndrome, Bolded P-value represents statistical significance

### Secretion of TrxR1 by T_reg_ and T_conv_ cells

In order to further confirm the changes in gene expression, we measured TrxR1 protein levels in supernatants from T_reg_ and T_conv_ cells collected from 32 subjects (18 CCS patients and 14 controls). The reason for measuring TrxT1 relied on a previous study by Xie et al. [25]. The Trx1 levels were generally low and almost undetectable in untreated samples. However, both T_reg_ and T_conv_ cells secreted significantly more TrxR1 after H_2_O_2_ treatment, p < 0.0001 in both cell types. Also, TrxR1 levels were significantly higher in supernatants from T_reg_ cells compared to T_conv_ cells, p = 0.003 (Fig. 2C). There were no differences between CCS patients and controls (Additional file 3: Figure S3).

### Correlations

There were significant inverse relationships between H_2_O_2_-induced intracellular ROS levels and % of living cells after 18 h, in particular for T_conv_ cells (Table 4). There was no correlation between intracellular ROS levels and STS-induced cell death. We were not able to study correlations between susceptibility to H_2_O_2_ ex vivo and antioxidant gene expression since these analyses were performed in different study subjects.

**Table 4:** Relationships between H_2_O_2_-induced intracellular ROS levels after 2 h treatment with 60 µM H_2_O_2_ and % of living cells after 18 h with increasing doses of H_2_O_2_ in regulatory T (T_reg_) cells and corresponding conventional T (T_conv_) cells

Treatment	T_reg_ cells	T_conv_ cells
5 µM H_2_O_2_	− 0.091	− 0.067
10 µM H_2_O_2_	− 0.422	− 0.571*
20 µM H_2_O_2_	− 0.336	− 0.609*
30 µM H_2_O_2_	− 0.530*	− 0.652**

Relationships are presented as Spearman correlation coefficients. *p < 0.05, **p < 0.01

TAC levels in freshly isolated PBMCs were measured in all study subjects and correlated inversely with intracellular ROS levels after 2 h treatment with 60 µM H_2_O_2_ in T_reg_ cells, *r* = − 0.480, *p* = 0.037; and T_conv_ cells, *r* = − 0.511, *p* = 0.030, but showed no correlations with H_2_O_2_-induced cell death ex vivo or with antioxidant gene expression.

## Discussion

The main findings from the present study are that human T_reg_ cells exhibit markedly increased sensitivity to H_2_O_2_-induced ROS production and cell death compared to T_conv_ cells in both CCS patients and healthy controls. T_reg_ cells were also more sensitive to spontaneous cell death when cultured in medium overnight and to cell death induced by STS, a potent inducer of apoptosis. Hitherto, the impact of oxidative stress on human T_reg_ cells has been far from clarified. The existing literature is both sparse and inconsistent. Mor et al. [16] reported that the number of T_reg_ cells was reduced to a considerably larger extent than the number of T_conv_ cells after in vitro incubation with oxidized LDL. They further showed that this effect was attenuated in the presence of caspase inhibitor suggesting that apoptosis contributed to the loss of cells. Several other studies have demonstrated that T_reg_ cells are more prone to apoptosis than T_conv_ cells when they are cultured in medium only [26–28]. On the other hand, Mougiakakos et al. [19] treated T cells with 5, 10 or 20 μM H_2_O_2_ for 18 h and found that T_reg_ cells were significantly more resistant to H_2_O_2_-induced cell death compared to T_conv_ cells. Moreover, they found that naïve T_reg_ cells were more resistant than memory T_reg_ cells. One possible explanation for the contradictory results may be the choice of study subjects. Mougiakakos et al. [19] used cells from a few healthy donors of unknown age while we used cells from sick and elderly subjects. Ageing and cardiovascular disease are both linked to elevated oxidative stress. We previously showed that naïve T_reg_ cells constituted only 15% of all T_reg_ cells in elderly CAD patients as opposed to 25% in age-matched controls, and also that the function of naïve as well as memory T_reg_ cells was impaired in patients, including lower suppressive capacity [9]. Differences in naïve T_reg_ cell pool size and function as well as individual variations in oxidative status may thus contribute to the disparity in results between our study and Mougiakakos et al. [19]. Still, important methodological aspects make it difficult to directly compare the studies. Mougiakakos et al. [19] used a different T_reg_ cell isolation protocol and they assessed cell death by labelling late apoptotic and necrotic cells, while we also included labelling of early apoptotic cells.

In an attempt to assess intrinsic ability of the cells to counteract oxidative stress, we measured the mRNA expression of several main antioxidant genes which all contribute to inactivate ROS, in freshly isolated T_reg_ and T_conv_ cells from both patients and controls. Collectively, the expression of catalase was significantly higher in T_reg_ cells than in T_conv_ cells and so was the expression of OXR1, a protein that has been described as a cellular oxidative stress sensor regulating the expression of several antioxidant enzymes [29]. Also, mRNA levels of the two main proteins, Trx and TrxR1, in the antioxidant Trx system were markedly upregulated in T_reg_ cells. Our findings partly agree with the previous study by Mougiakakos et al. [21], who reported that Trx expression was higher in T_reg_ cells compared with T_conv_ cells while, on the other hand, catalase expression did not differ in their small study group of volunteers. An earlier genomic and proteomic screening study examined the H_2_O_2_-induced gene and protein expression in human skin fibroblasts and reported that TrxR1 was the only oxidation-related candidate with elevated levels at both the mRNA and protein level, the latter measured in cell lysates [25]. Another study by Söderberg et al. [30] provided evidence that TrxR1 was secreted by human PBMCs upon inflammatory stimulation. Here, we were able to show that TrxR1 was secreted into the cell supernatant to a greater extent by T_reg_ cells than by T_conv_ cells upon H_2_O_2_ treatment. Altogether, our findings indicate that T_reg_ cells have a higher endogenous antioxidant capacity than T_conv_ cells in both CCS patients and controls.

Several studies have reported reduced levels of T_reg_ cells in peripheral blood of patients with ACS [9, 15–17]. It has also been proposed that oxidative stress is involved in the depletion of T_reg_ cells. Mor et al. [16] showed that T_reg_ cells from ACS patients were more sensitive to oxidized LDL than T_reg_ cells from CCS patients or patients with normal coronary angiograms. A later study by Zhang et al. [17] showed that the spontaneous apoptosis of T_reg_ cells was pronounced in ACS patients and further demonstrated that oxLDL was able to induce apoptosis of human T_reg_ cells in vitro, yet without comparing T_reg_ and T_conv_ cells. Recently, we found that the numerical and functional T_reg_ cell deficit in ACS patients was not merely a transient phenomenon but that it remained after clinical stabilization [9]. In the present study, we therefore focused on the potential role of oxidative stress in the depletion of T_reg_ cells in CCS patients. However, there was no proof that T_reg_ cells from patients were more prone to H_2_O_2_-induced cell death than T_reg_ cells from controls, when assessed ex vivo. Moreover, the expression of antioxidant genes in freshly isolated T_reg_ cells from patients and controls indicated that T_reg_ cells in CCS patients and controls had similar levels of endogenous antioxidant enzymes in vivo. However, the significantly lower intrinsic TAC levels in PBMC from CCS patients compared with PBMC from controls pointed toward an oxidant-antioxidant imbalance in patients, highlighting the possible impact of exogenous environmental factors, such as sedentary lifestyle and unhealthy diet. The TAC assay is a copper-based assay which measures a large range of lipophilic or thiol-based antioxidants and antioxidant macromolecules but not the antioxidant enzymes discussed above [31, 32]. As a further evidence of oxidant-antioxidant imbalance, we found that the oxLDL/LDL ratios were significantly higher in the patient group indicating a higher degree of oxdation in their LDL particles. Therefore, given the high susceptibility of T_reg_ cells to oxidative stress, it is reasonable to assume that the numbers of T_reg_ cells may be affected by the prooxidant state in CCS patients.

Interestingly, our results indicate that T_conv_ cells from CCS patients were affected to a greater extent by oxidative stress than T_conv_ cells from controls. T_conv_ cells from CCS patients were more prone to undergo cell death when treated with H_2_O_2_ or STS and they also expressed significantly higher levels of TrxR1. It is well documented that the proatherogenic T cell response in atherosclerotic lesions is reflected in peripheral blood [8]. In ACS patients, systemic T cell activation is associated with plaque instability and thrombus formation but there is also consistent evidence that the T cell activation persists after clinical stabilization [8–10]. T cell activation is accompanied by the release of ROS which in turn leads to an upregulation of endogenous antioxidants [33]. Moreover, the induction of Trx and TrxR1 in T cells upon activation was recently suggested to be a critical pathway controlling T cell activation and expansion [34]. We believe that the increased susceptibility to oxidative stress in T_conv_ cells from patients reflects a CD4^+^ T cell activation that remains in CCS patients despite clinical stability and medical treatment.

A couple of limitations should be considered in this pilot study. One limitation is the limited number of patients and controls per experimental set-up, permitting only cautious conclusions about differences between the two groups. The yield levels of T_reg_ cells in the subjects, particularly in the CCS patients, were not high enough to allow ex vivo experiments and gene expression analyses to be carried out in the same individuals, nor to perform functional assays. Gene expression analyses were therefore performed in a separate group of subjects, 10 patients and 10 controls, though with similar characteristics as the other subjects. Another potential limitation is that the ex vivo model, where isolated fractions of T cell subsets are exposed to increasing doses of H_2_O_2_ for a relatively short period of time, may not reflect chronic inflammation.

## Conclusion

T_reg_ cells were highly susceptible to oxidative stress and cell death ex vivo. T_reg_ cells also expressed and secreted high levels of antioxidants indicating a high endogenous capacity to counteract oxidative stress in both CCS patients and controls. Given the presence of oxidant-antioxidant imbalance in the patient group, we postulate that oxidative stress may be a contributor to the T_reg_ cell deficit in vivo.

## Supplementary Information

**Additional file 1: Figure S1**. Representative flow cytometry images of T_reg_ cells defined as CD4^+^CD127^low^CD25^+^ (A) and T_conv_ cells defined as CD4^+^CD127^+^CD25^−^ T cells (B) sorted with the EasySep Human CD4^+^CD127^low^CD25^+^ Regulatory T Cell Isolation Kit. Characteristics shown are from left to right: forward and side scatter, CD4^+^ signal, CD25^+^ signal, and CD127^+^ signal. The expression of Foxp3 in T_reg_ and T_conv_ cells sorted with the EasySep Human CD4^+^CD127^low^CD25^+^ Regulatory T Cell Isolation Kit is shown in Figure C.

**Additional file 2: Figure S2**. Representative images for assessing sensitivity to ROS-induced cell death. The results from isolated T_reg_ and T_conv_ cells are shown from the left to the right; untreated, treated with 10 µM H_2_O_2_ or treated with 30 µM H_2_O_2_. Annexin V and SYTOX signals are shown on the x and y axis, respectively. Regions include double-negative (S^−−^), Annexin V positive (S^+−^), SYTOX positive (S^−+^), and double-positive (S^++^). Since it is difficult to completely separate the transition from apoptosis to necrosis with this method, cells negative for both Annexin-V and SYTOX were considered viable.

**Additional file 3: Figure S3.** The amount of oxidative stress, measured as mean fluorescence intensity (MFI) of CellROX Green, in (A) T_reg_ cells from CCS patients and controls and (B) T_conv_ cells from CCS patients and controls following treatment with 0, 10, 30, 60 or 120 µM H_2_O_2_ for 2 h.

## Data Availability

All datasets used and analyzed throughout the study are available from the corresponding author based on sensible request.
