# Supplementary figures and images for: Oxidative stress response in regulatory and conventional T cells: a comparison between patients with chronic coronary syndrome and healthy subjects
Source: J Transl Med. 2021 Jun 3;19:241. doi: 10.1186/s12967-021-02906-2 (PMC8173731; doi:10.1186/s12967-021-02906-2)

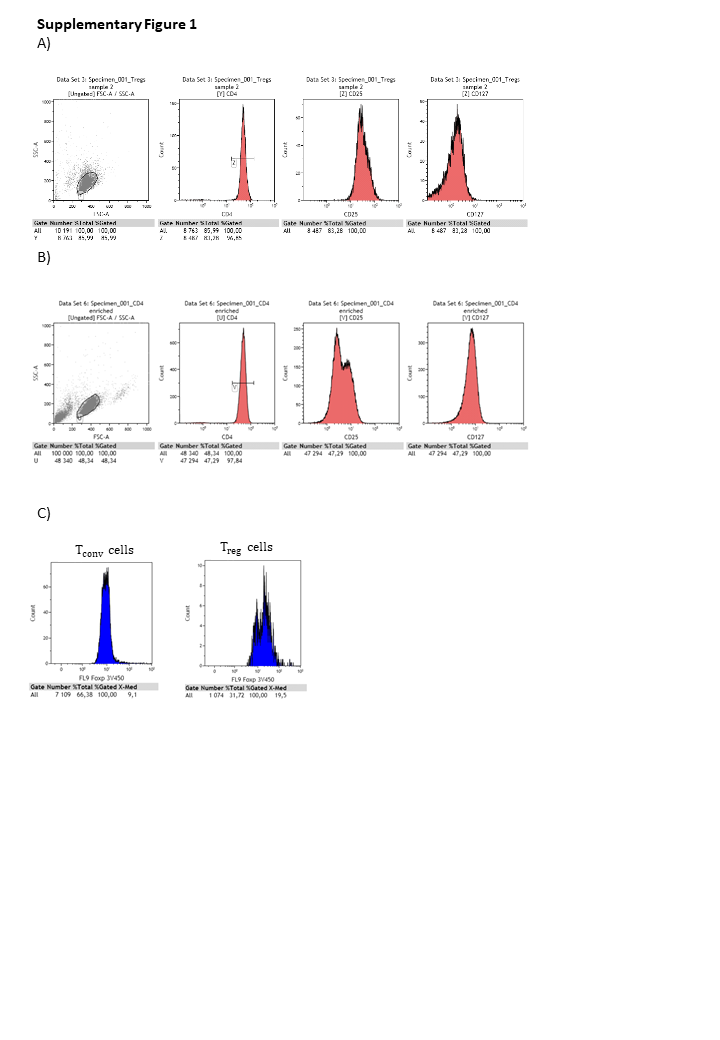

Supplement: Supplementary file 1 — Additional file 1: Figure S1. Representative flow cytometry images of Treg cells defined as CD4+CD127lowCD25+ (A) and Tconv cells defined as CD4+CD127+CD25− T cells (B) sorted with the EasySep Human CD4+CD127lowCD25+ Regulatory T Cell Isolation Kit. Characteristics shown are from left to right: forward and side scatter, CD4+ signal, CD25+ signal, and CD127+ signal. The expression of Foxp3 in Treg and Tconv cells sorted with the EasySep Human CD4+CD127lowCD25+ Regulatory T Cell Isolation Kit is shown in Figure C. [file 12967_2021_2906_MOESM1_ESM.tiff]

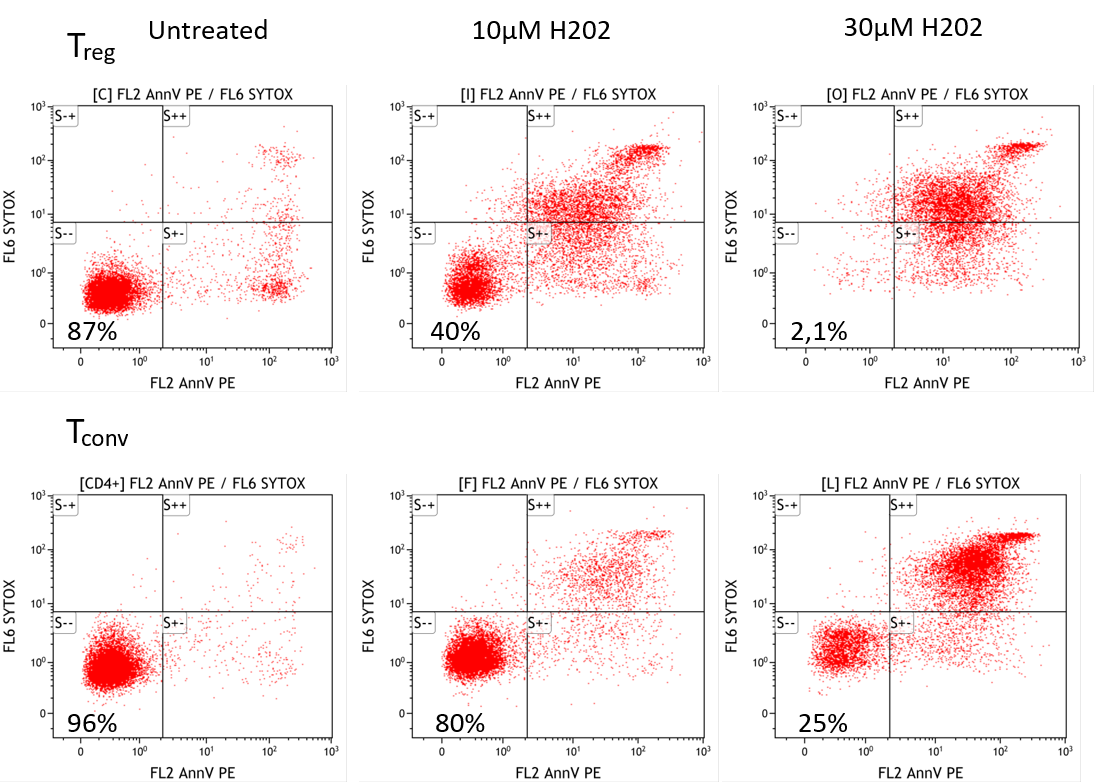

Supplement: Supplementary file 2 — Additional file 2: Figure S2. Representative images for assessing sensitivity to ROS-induced cell death. The results from isolated Treg and Tconv cells are shown from the left to the right; untreated, treated with 10 µM H2O2 or treated with 30 µM H2O2. Annexin V and SYTOX signals are shown on the x and y axis, respectively. Regions include double-negative (S−−), Annexin V positive (S+−), SYTOX positive (S−+), and double-positive (S++). Since it is difficult to completely separate the transition from apoptosis to necrosis with this method, cells negative for both Annexin-V and SYTOX were considered viable. [file 12967_2021_2906_MOESM2_ESM.tiff]

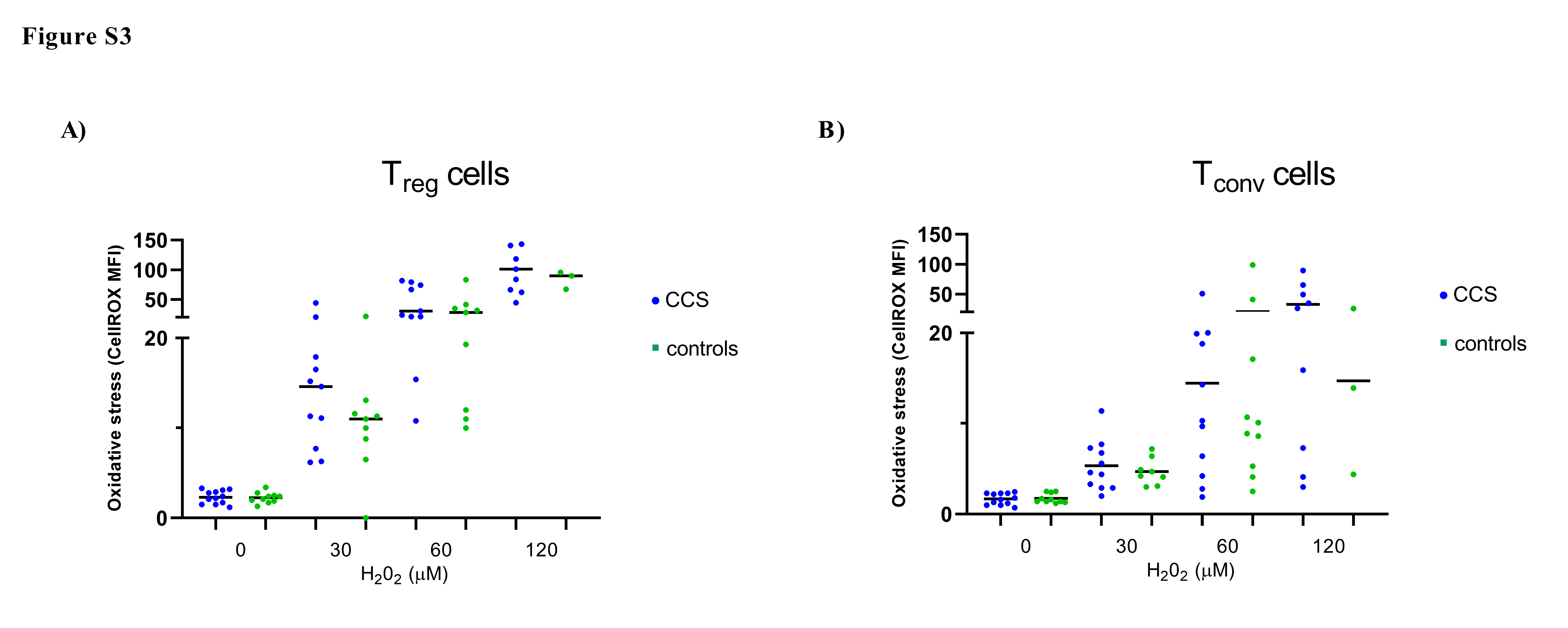

Supplement: Supplementary file 3 — Additional file 3: Figure S3. The amount of oxidative stress, measured as mean fluorescence intensity (MFI) of CellROX Green, in (A) Treg cells from CCS patients and controls and (B) Tconv cells from CCS patients and controls following treatment with 0, 10, 30, 60 or 120 µM H2O2 for 2 h. [file 12967_2021_2906_MOESM3_ESM.tiff]
